# Phylogenetic insight into subgenera *Idaeobatus* and *Malachobatus* (*Rubus*, Rosaceae) inferring from ISH analysis

**DOI:** 10.1186/s13039-015-0114-y

**Published:** 2015-02-03

**Authors:** Yan Wang, Xiaorong Wang, Qing Chen, Li Zhang, Haoru Tang, Ya Luo, Zejing Liu

**Affiliations:** College of Horticulture, Sichuan Agricultural University, Ya’an, 625014 People’s Republic of China; Institute of Pomology and Olericulture, Sichuan Agricultural University, Chengdu, 611130 People’s Republic of China

**Keywords:** *Rubus*, Allopolyploid, Hybrid, rDNA-FISH, GISH

## Abstract

**Background:**

*Rubus* is a large and taxonomically complex genus exhibiting agamospermy, polyploidy and frequent hybridization. The objective of this work was to elucidate rDNA disrtibution pattern and investigate genomic composition of polyploids in 16 *Rubus* taxa (2*n* = 2*x*, 3*x*, 4*x*, 8*x*) of two subgenera *Idaeobatus* and *Malachobatus* by ISH method.

**Results:**

The basic *Rubus* genome had one 45S rDNA locus, and all the polyploids (except *R. setchuenensis*) had the expected multiples of this number. Diploid and tetraploid *Rubus* taxa carried two 5S rDNA, whereas the triploid and octoploid species only had three. The duplicated 45S rDNA sites tended to be conserved, whereas those of 5S rDNA tended to be eliminated after polyploidization. The accession *R03-20* was an autotriploid *R. parvifolius*, while *R03-27* and *R03-57* were naturally-occurred triploid hybrids between *R. parvifolius* and *R. coreanus*. GISH results suggested that *R. parvifolius* had close relationship with polyploids from *Malachobatus*.

**Conclusions:**

The polyploids from *Malachobatus* were probable allopolyploid. In addition, *Rubus parvifolius* might be involved in hybridization, polyploidization and speciation of some *Idaeobatus* and *Malachobatus* species.

## Background

*Rubus* Linnaeus (Rosaceae) has long been deemed taxonomically challenging due to its propensity for agamospermy, polyploidy and frequent hybridization [1]. This genus is divided into 12 subgenera, with numerous infrageneric sections and series [2-4], containing several hundreds of sexual species to perhaps thousands of apomictic microspecies [5-7]. China, exceptionally rich in *Rubus* species, especially the south-western part of the country, was proposed to be a major centre of diversity for the genus, with about 200 species [8]. Most species here are mainly concentrated in two subgenera (or sections by Lu and Yü et al.), *Idaeobatus* and *Malachobatus*, estimated as much as 83 and 84 species, respectively, containing 24 sections [9,10]. Subgenus *Idaeobatus* species are predominantly diploid, whereas subg. *Malachobatus* represents a polyploid complex, with tetraploidy, hexaploidy, octoploidy or tetradecaploidy level [6,11,12]. Interestingly, *R. parvifolius* in subg. *Idaeobatus* had various ploidy levels, with di-, tri-, tetraploid, and mixed diploid-tetraploid [13]. Not only are the phylogenetic relationships between these species unknown, we also don’t even know to what extent ploidy level varies among them.

Evolutionary process in the genus *Rubus* has been argued for a long time. Based on data from morphology and chromosome counts, Lu [8] suggested that evolution in *Rubus* proceeded from woody to herbaceous plants, and from compound to simple leaves. If Lu’s morphological hypothesis [8] was correct, then species in subg. *Idaeobatus* might be the most primitive with woody plant and compound leaf, and members of subg. *Malachobatus* were more advanced with simple-leaved species. However, molecular phylogenetic evidence from ITS markers did not seem to support this hypothesis [14]. Recently, Alice et al. [15], with ITS, *rpl*16, and *trn*L/F sequences, proposed the possibility that subg. *Malachobatus* was originated from subg. *Idaeobatus* diploid species but failed to seek out these ancestors in his study. In addition, previous studies using both ITS and cpDNA (*ndh*F, *rpl*16 and *trn*L/F) sequences from Bhutanese, Korean, and Pacific *Rubus* indicated that the subg. *Idaeobatus* was a polyphyletic group, forming at least two distinct groups [14,16,17], implying that subg. *Idaeobatus* might be relative primitive in the genus *Rubus*. In comparative studies on karyotype of 28 taxa from subg. *Idaeobatus* and *Malachobatus*, eighteen taxa from 7 sections of subg. *Idaeobatus* showed diverse chromosome morphology both between and within sections, while species belonging to 6 sections of subg. *Malachobatus* exhibited uniform intra-sectional karyotypic features but different karyotypes between sections. As a result, we inferred that species of subg. *Idaeobatus* with abundant genetic variation were of more complex taxon than those of *Malachobatus* [12]. These facts indicated that some particular subg. *Idaeobatus* species*,* being involved in hybridization, polyploidization and speciation of *Rubus*, might play an important role in phylogeny of the genus. It has been reported that some *Idaeobatus* species can hybridize each other freely and produce fertile offspring, e.g., *R. parvifolius* with *R. coreanus* and *R. sieboldii*; *R. trifidus* with *R. hirsutus*, *R. microphyllus* and *R. palmatus* [18-23].

*Rubus parvifolius* and *R. coreanus*, are two widely distributed species of subg. *Idaeobatus* in China [10,24]. In our field investigation, *R. parvifolius* displays remarkable morphological diversity in traits such as leaf size, prickle density, fruit size, and seed number. There are also some differences in the color of canes and prickles, most of which were reddish brown but a few were green. Moreover, *R. parvifolius* exhibited not only abundant molecular variation, but also various ploidy levels and conspicuous different karyotypes (with 2*x*, 3*x*, 4*x*, and cytotype mixture of 2*x* and 4*x*), whereas another widely-distributed species *R. coreanus* revealed typical morphology and highly uniform ploidy (2*x*) and karyotype [13]. Interestingly, both two species often grow sympatrically with those species from both *Idaeobatus* and *Malachobatus*. It has been reported that *R. parvifolius* could facilitate natural hybridization and formations of natural hybrids with *R. coreanus* (*R. × hiraseanus*, 2*x* and 3*x*) and *R. phoenicolasius* (*R. × nikaii*, 2*x*) from *Idaeobatus* [18,22,23], and *R. seiboldii* (*R. × tawadanus*, 3*x*) from *Malachobatus* [19,21]. There has not any report that *R. coreanus* could hybridize with other *Rubus* species other than *R. parvifolius* yet. Therefore, we speculated that *R. parvifolius* might play an important role in speciation and phylogeny in both subgenera *Idaeobatus* and *Malachobatus* in *Rubus* genus.

*In situ* hybridization techniques are effective tools for phylogenetic inference and hybrid identification in plant research. Fluorescence *in situ* hybridization (FISH) has been used for physical mapping of repetitive DNA sequences and multi-copy families [25,26]. It is possible to determine the genomic homology between species and identify chromosome composition of hybrids by genomic *in situ* hybridization (GISH) [25,27]. Previous studies on cytogenetics on *Rubus* were mostly limited to chromosome counting [6,11,19,20,28-31] or traditional karyotype analysis [12,13,18,21-23,32,33]. FISH with 45S rDNA was established in *R. parvifolius* [34] and callus lines from European and American countries [35]. Screening power of GISH in identifying interspecies hybrids of raspberry and blackberry had already been demonstrated [36,37]. These molecular techniques were expected to be applied to phylogenetic analysis among other *Rubus* species.

In this study, we tried to obtain new phylogenetic insight into the subgenera *Idaeobatus* and *Malachobatus* of *Rubus*, and seek for any clues to infer the speculated role of *R. parvifolius* in speciation and polyploidization processes of the genus. Physical distribution patterns of 45S and 5S rDNA in 18 accessions, including 14 *Rubus* taxa (2*n* = 2*x*, 4*x*, 8*x*) and 3 resembling *R. parvifolius* accessions (2*n* = 3*x*), from 9 out of 24 sections belonging to the two subgenera were analyzed by FISH. Comparative GISH analysis on these taxa probed with *R. parvifolius* DNA (as *R. coreanus* a comparison) was conducted. In addition, three triploid accessions: *R03-20* (recently collected), *R03-27* [38] and *R03-57* (recently collected) were included here to identify their genomic composition. All these results will shed light on the phylogenetic history of the genus *Rubus*.

## Results

### Number and localization of 45S and 5S rDNA sites

**Figure 1:**
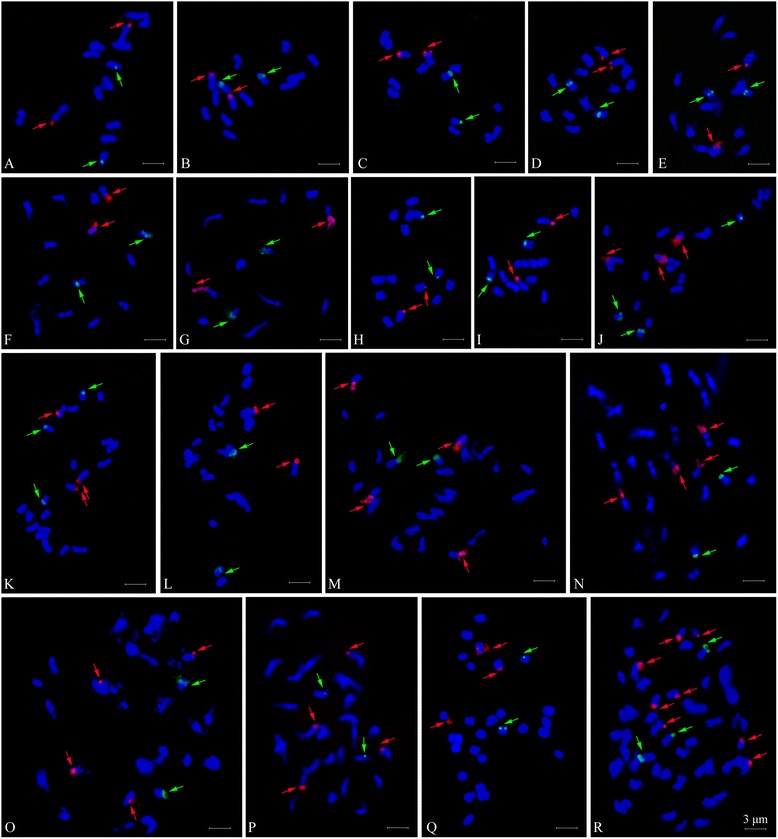
**Numbers of 45S (red) and 5S rDNA (green) sites visualized by FISH in eighteen accessions from subgenera** ***Idaeobatus*** **and** ***Malachobatus*** **of the genus** ***Rubus*** **. A**: *R. niveus*; **B**: *R. ellipticus*; **C**: *R. ellipticus* var. *obcordatus*; **D**: *R. parvifolius*; **E**: *R. pinfaensis*; **F**: *R. coreanus* (*R01-4*); **G**: *R. coreanus* (*R03-6*); **H**: *R. tsangii*; **I**: *R. corchorifolius*; **J**: *R03-20*; **K**: *R03-27*; **L**: *R03-57*; **M**: *R. lambertianus* var. *glaber*; **N**: *R. parkeri*; **O**: *R. assamensis*; **P**: *R. multibracteanus*; **Q**: *R. setchuenensis*; **R**: *R. buergeri*.

**Figure 2:**
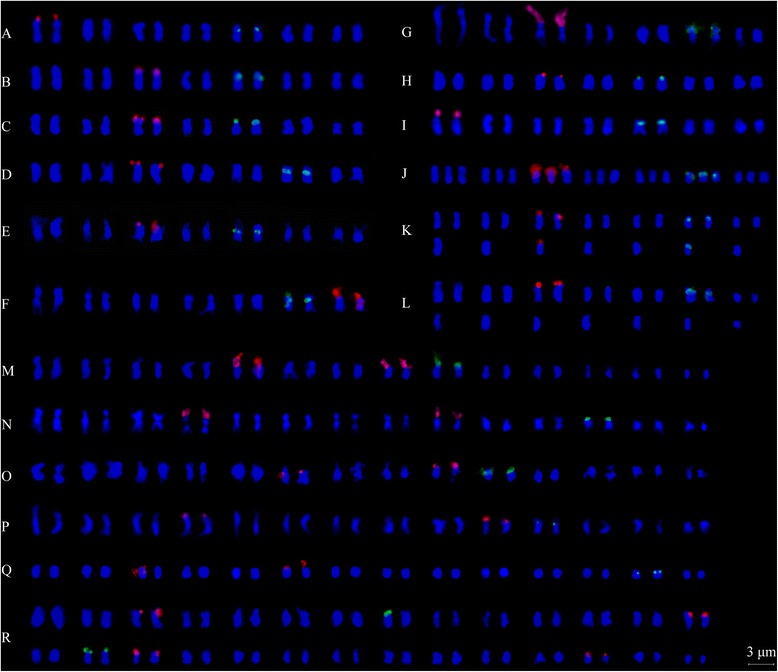
**Karyotype of eighteen** ***Rubus*** **accessions revealed by FISH. A**: *R. niveus*; **B**: *R. ellipticus*; **C**: *R. ellipticus* var. *obcordatus*; **D**: *R. parvifolius*; **E**: *R. pinfaensis*; **F**: *R. coreanus* (*R01-4*); **G**: *R. coreanus* (*R03-6*); **H**: *R. tsangii*; **I**: *R. corchorifolius*; **J**: *R03-20*; **K**: *R03-27*; **L**: *R03-57*; **M**: *R. lambertianus* var. *glaber*; **N**: *R. parkeri*; **O**: *R. assamensis*; **P**: *R. multibracteanus*; **Q**: *R. setchuenensis*; **R**: *R. buergeri*.

In either diploid or polyploid species, 45S rDNA sites localized at the terminal regions, and 5S rDNA sites were localized on proximal or sub-terminal region of the short arms (Figures 1 and 2, Table 1). Nine diploid taxa exhibited two 45S and two 5S rDNA sites, despite variable signal intensity (Figures 1 and 2A-I), in congruent with their diploid levels. A pair of satellites was detected along with two strong 45S rDNA sites in both *R. niveus* (*R01-1*) and *R. corchorifolius* (*R01-6*) (Figures 1 and 2A and I). The three taxa, *R. ellipticus* (*R01-7*), *R. ellipticus* var. *obcordatus* (*R01-2*), and *R. pinfaensis* (*R01-22*), had two 45S rDNA sites on terminal chromosomal regions of chromosome 3. 5S rDNA sites localized on the proximal region of short arms of chromosome 5 in *R. ellipticus* and *R. pinfaensis*, but existed at sub-terminal regions of chromosome 5 in *R. ellipticus* var. *obcordatus* (Figures 1 and 2B,C and E). There was an identical 45S and 5S rDNA distribution of chromosome 3 and 6 in *R. parvifolius* (*R03-5*) and *R. coreanus* (*R03-6*) (Figures 1 and 2D and G) from Xichong county. In contrast, *R. coreanus* (*R01-4*) from Ya’an city had two 45S rDNA sites distributing on the satellites of chromosome 7 and two 5S rDNA sites at the proximal region of the short arm on chromosome 6 (Figures 1 and 2F).

**Table 1:** **Numbers of 45S and 5S rDNA sites and GISH signals on chromosomes of these accessions tested in** ***Rubus***

**Taxa**	**2** ***n***	**Locality**	**Voucher**	**rDNA loci number and distribution**			**GISH signals on chromosomes probed with** ***R. parvifolius*** **(** ***R03-5*** **, 2** ***x*** **)**	
				**45S**	**5S**	**Figures** **1** **and** **2**	**Number and Localization**	**Figures** **3** **and** **4**
**Subg.** ***Idaeobatus*** **Focke**								
Sect. *Idaeanthi* (Focke) Yü & Lu								
*R. niveus* Thunb.	14	Ya’an, Sichuan	X.R. Wang *R01-1*	2 (1S^SAT)^	2 (5S^P^)	A	8 (2S^PC^, 4S^PC^, 6S^PC^, 7S^PC^)	A
Sect. *Stimulantes* Yü & Lu								
*R. ellipticus* Smith	14	Ya’an, Sichuan	X.R. Wang *R01-7*	2 (3S^T^)	2 (5S^P^)	B	14 (Dispersed through chromosomes)	B
*R. ellipticus* Smith var. *obcordatus* (Franch.) Focke	14	Ya’an, Sichuan	X.R. Wang *R01-2*	2 (3S^T^)	2 (5S^ST^)	C	14 (3S^T^, 1-2S^PC^, 4-7S^PC^)	C
*R. parvifolius* L.	14	Xichong, Sichuan	X.R. Wang *R03-5*	2 (3S^T^)	2 (6S^P^)	D	14 (3S^T^, 1-2S^PC^, 4-7S^PC^)	D
Sect. *Pungentes* (Focke) Yü & Lu								
*R. pinfaensis* Lévl. Et Vant.	14	Ya’an, Sichuan	X.R. Wang *R01-22*	2 (3S^T^)	2 (5S^P^)	E	14 (Dispersed through chromosomes)	F
*R. coreanus* Miq.	14	Ya’an, Sichuan	X.R. Wang *R01-4*	2 (7S^SAT^)	2 (6S^P^)	F	—	—
	14	Xichong, Sichuan	X.R. Wang *R03-6*	2 (3S^SAT^)	2 (6S^P^)	G	14 (3S^T^, 1-7S^PC^)	G
Sect. *Rosaefolii* (Focke) Yü & Lu								
*R. tsangii* Merr.	14	Ya’an, Sichuan	X.R. Wang *R01-21*	2 (3S^T^)	2 (5S^ST^)	H	8 (1S^T^, 3S^T^, 4S^T^,6S^T^)	H
Sect. *Corchorifolii* (Focke) Yü & Lu								
*R. corchorifolius* L. f.	14	Ya’an, Sichuan	X.R. Wang *R01-6*	2 (1S^SAT^)	2 (5S^P^)	I	6 (Dispersed through chromosomes 2, 4 and 5)	I
Triploid accessions	21	Xichong, Sichuan	X.R. Wang *R03-20*	3 (3S^T^)	3 (6S^P^)	J	21 (15 in S^PC^, 6 dispersed through chromosomes)	E
	21	Xichong, Sichuan	X.R. Wang *R03-27*	3 (3S^T^)	3 (6S^P^)	K	—	—
	21	Xichong, Sichuan	X.R. Wang *R03-57*	2 (3S^T^)	2 (6S^P^)	L	—	—
**Subg.** ***Malachobatus*** **Focke**								
Sect. *Acuminati* (Focke) Yü & Lu								
*R. lambertianus* Ser. var. *glaber* Hemsl.	28	Ya’an, Sichuan	X.R. Wang *R01-8*	4 (5S^T^, 8S^T^)	2 (9S^ST^)	M	14 (5S^T^, 9S^T^, other dispersed through chromosomes 1, 3, 6 and 8)	J
Sect. *Dolichophylli* Yü & Lu								
*R. parkeri* Hance	28	Ya’an, Sichuan	X.R. Wang *R01-12*	4 (4S^T^, 9S^T^)	2 (12S^ST^)	N	8 (1S^T^, 6S^T^, 9S^T^, 11S^T^)	K
Sect. *Elongati* (Focke) Yü & Lu								
*R. assamensis* Focke	28	Ya’an, Sichuan	X.R. Wang *R01-10*	4 (6S^T^, 9S^T^)	2 (10S^P^)	O	4 (4S^T^, 9S^T^)	L
Sect. *Moluceani* (Focke) Yü & Lu								
*R. multibracteatus* Lévl. Et Vant.	28	Ya’an, Sichuan	X.R. Wang *R01-23*	4 (4S^T^, 10S^T^)	2 (11S^ST^)	P	12 (1S, 3S^PC^, 4S^T^, 10S^T^, others dispersed through chromosomes 7 and 8)	M
*R. setchuenensis* Bureau et Franch.	28	Ya’an, Sichuan	X.R. Wang *R01-24*	3 (3S^T^, 6S^T^)	2 (13S^P^)	Q	6 (3S^T^, 6S^T^, 11S^T^)	N
*R. buergeri* Miq.	56	Ya’an, Sichuan	X.R. Wang *R01-11*	8 (3S^T^, 14S^T^, 17S^T^, 26S^T^)	3 (8S^ST^, 16S^ST^)	R	2 (14S^T^)	O

Note: The classification system follows that of Focke (1910, 1911, 1914). L: long arm; S: short arm; SAT: satellite; T: terminal region; ST: sub-terminal region; P: proximal region; PC: peri-centromeric region.

As for the three triploid accessions, three hybridization signals with the same intensity were detected using both 45S and 5S rDNA probes in *R03-20* (Figures 1 and 2J). Accession *R03-27* exhibited three 45S and three 5S rDNA sites, with one 5S rDNA signal was much larger and more intensive than the other two (Figures 1 and 2K). However, only two 45S and two 5S rDNA sites were found in *R03-57*, both signals the same intensity, locating on the chromosome 3 and 6, respectively (Figures 1 and 2L).

The four tetraploid taxa, *R. lambertianus* var. *glaber* (*R01-8*), *R. parkeri* (*R01-12*), *R. assamensis* (*R01-10*), and *R. multibracteatus* (*R01-23*), shared the same number of rDNA sites, four for 45S rDNA and two for 5S rDNA sites (Figures 1 and 2M-P). The number of 45S rDNA was twice to that detected in diploids. Although the number and the position of rDNA sites were strictly consistent among the tetraploids, variations in signal size and intensity among sites were observed. For instance, two 45S rDNA loci presented remarkably higher levels of signal intensity than the rest in the four species (Figures 1 and 2M-P). However, only three 45S rDNA signals were detected in *R. setchuenensis* (*R01-24*), one signal being significantly larger than the other two (Figures 1 and 2Q). Two 5S rDNA sites showed equal intensities on a pair of chromosomes in five tetraploids (Figures 1 and 2M-Q).

In octoploid *R. buergeri* (*R01-11*) (Figures 1 and 2R), eight 45S rDNA sites were detected, among which, two were markedly bigger than the others. Only three 5S rDNA sites were found in *R. buergeri*, much less than the number we anticipated based on comparison with the diploids.

### GISH analysis of fourteen accessions from *Idaeobatus* and *Malachobatus* probed with *R. parvifolius*

**Figure 3:**
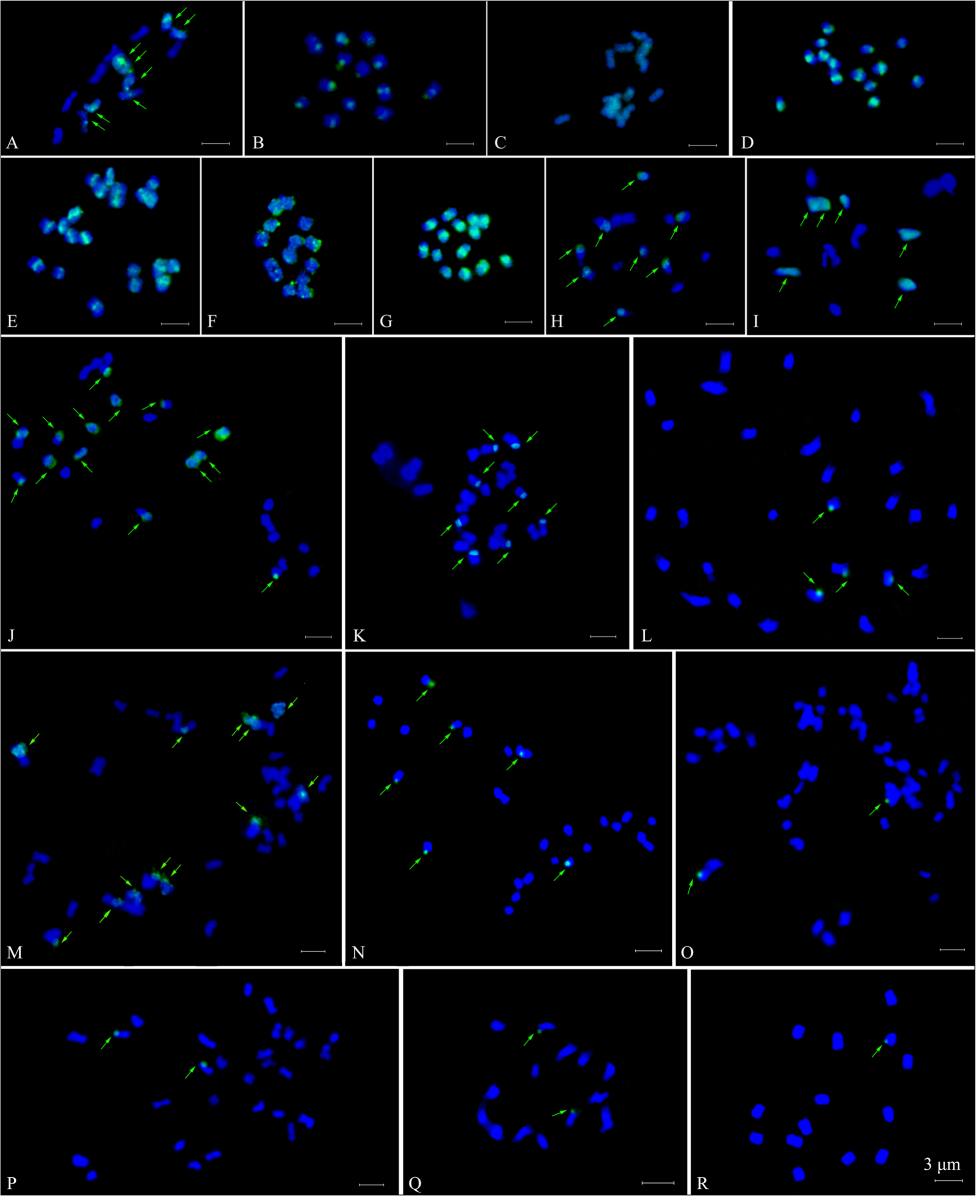
**GISH signal patterns on chromosomes of fifteen** ***Rubus*** **accessions (A-O) from subgenera** ***Idaeobatus*** **and** ***Malachobatus*** **probed by** ***R. parvifolius*** **(** ***R03-5*** **; 2** ***x*** **) DNA. A**: *R. niveus*; **B**: *R. ellipticus*; **C**: *R. ellipticus* var. *obcordatus*; **D**: *R. parvifolius*; **E**: *R03-20*; **F**: *R. pinfaensis*; **G**: *R. coreanus* (*R03-6*); **H**: *R. tsangii*; **I**: *R. corchorifolius*; **J**: *R. lambertianus* var. *glaber*; **K**: *R. parkeri*; **L**: *R. assamensis*; **M**: *R. multibracteatus*; **N**: *R. setchuenensis*; **O**: *R. buergeri*. **P**: GISH signals in *R. assamensis* (*R01-10*, 4*x*) probed with *R. coreanus* (*R03-6,* 2*x*). **Q-R**: GISH results in *R. parvifolius* (*R03-5*, 2*x*) and *R. coreanus* (*R03-6*, 2*x*) probed by *R. assamensis* (*R01-10*, 4*x*), respectively.

**Figure 4:**
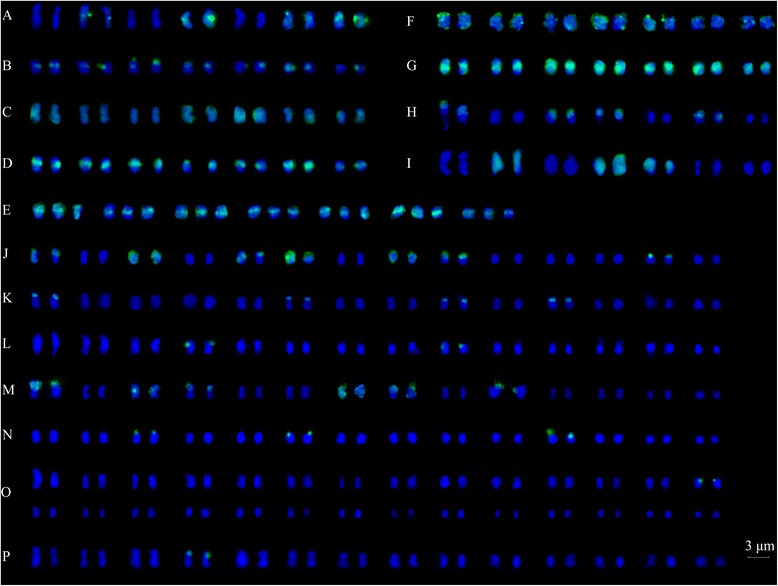
**Karyotype of fifteen** ***Rubus*** **accessions (A-O) revealed by GISH analysis probed by DNA of** ***R. parvifolius*** **(** ***R03-5*** **; 2** ***x*** **). A**: *R. niveus*; **B**: *R. ellipticus*; **C**: *R. ellipticus* var. *obcordatus*; **D**: *R. parvifolius* (*R03-5*); **E**: *R03-20*; **F**: *R. pinfaensis*; **G**: *R. coreanus*; **H**: *R. tsangii*; **I**: *R. corchorifolius*; **J**: *R. lambertianus* var. *glaber*; **K**: *R. parkeri*; **L**: *R. assamensis*; **M**: *R. multibracteatus*; **N**: *R. setchuenensis*; **O**: *R. buergeri*. **P**: GISH signals in *R. assamensis* (*R01-10*, 4*x*) probed with *R. coreanus* (*R03-6*, 2*x*).

It was obvious that at ~85% stringency, the *parvifolius* (*R03-5*; 2*x*) DNA probe generated a large number of signals dispersedly distributed through six to fourteen chromosomes in all diploid species (Figures 3 and 4A-D, F-I). The all chromosomes of *R03-20* (3*x*) and *R. coreanus* (*R03-6*) displayed the strongest signal intensity at the centromeric and terminal regions (Figures 3 and 4E and G). This was similar to the signal pattern of self-GISH in *R. parvifolius* (Figures 3 and 4D). GISH results also revealed that fourteen chromosomes of *R. ellipticus* (*R01-7*), *R. ellipticus* var. *obcordatus* (*R01-2*) and *R. pinfaensis* (*R01-22*) were weakly labelled at the same regions (Figures 3 and 4B, C and F). The hybridization signals were detected at the same regions on eight chromosomes of *R. niveus* (*R01-1*) and *R. tsangii* (*R01-21*) (Figures 3 and 4A and H). The remaining species, *R. corchorifolius* (*R01-6*) exhibited weak signals at the centromeric or telomeric region on six chromosomes (Figures 3 and 4I).

Among the five tetraploids, fourteen chromosomes of *R. lambertianus* var. *glaber* (*R01-8*) were labelled with genomic DNA of *R. parvifolius*, eight at the telomeric regions, four through along the chromosomes, and two at the centromeric parts (Figures 3 and 4J). Eight and twelve chromosomes were labelled clearly at telomeric and centromeric regions of *R. parkeri* (*R01-12*) and *R. multibracteatus* (*R01-23*), respectively (Figures 3 and 4K and M). In *R. assamensis* (*R01-10*) and *R. setchuenensis* (*R01-24*), four and six signals were detected at the telomeric parts (Figures 3 and 4L and N). Only two weak signals were detected at the telomeric regions in octoploid *R. buergeri* (*R01-11*) (Figures 3 and 4O).

When using probe from another widely distributed species *R. coreanus* (*R03-6*), only two signals were observed in tetraploid species (one taxon *R. assamensis* was shown in Figure 3P). Only two or one signal in *R. parvifolius* or *R. coreanus* was detected using genomic DNA of the polyploids as probes (only data for two representative taxa were shown here, in Figure 3Q and R).

### GISH analysis of two triploids probed with *R. parvifolius* and *R. coreanus*

**Figure 5:**
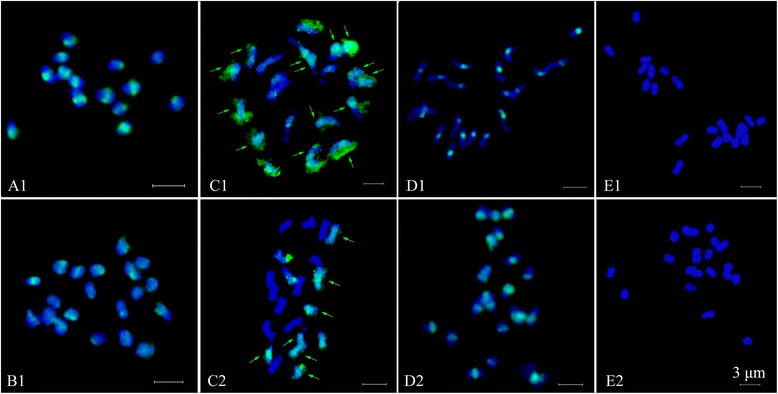
**GISH results on chromosomes of** ***R03-5*** **(2** ***x*** **, A1),** ***R03-20*** **(3** ***x*** **, B1), and** ***R03-27*** **(3** ***x*** **, C-E) probed by DNA from** ***R. parvifolius*** **(1:** ***R03-5*** **) and** ***R. coreanus*** **(2:** ***R03-6*** **).** The ratio of blocking DNA to probe DNA was 50 **(C)**, 0 **(D)**, and 100 **(E)**, respectively.

**Figure 6:**
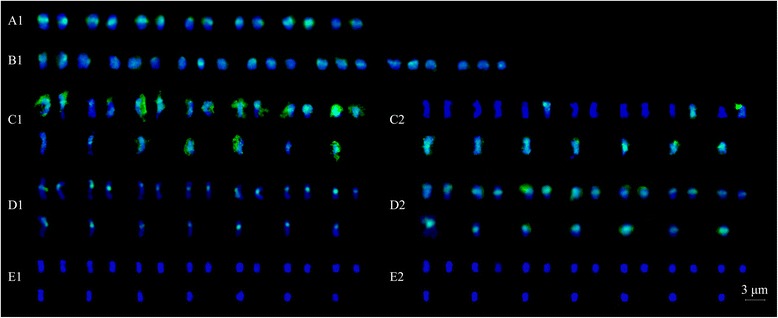
**Karyotype of** ***R03-5*** **(2** ***x*** **, A1),** ***R03-20*** **(3** ***x*** **, B1), and** ***R03-27*** **(3** ***x*** **, C-E) based on GISH results probed by DNA from** ***R. parvifolius*** **(1:** ***R03-5*** **) and** ***R. coreanus*** **(2:** ***R03-6*** **).**

Self-GISH in *R. parvifolius*, the pericentromeric regions of all mitotic chromosomes were strongly labelled, whereas other chromosomal regions were not (Figures 5 and 6A1). 21 chromosomes of *R03-20* showed strong signals at centromeric parts when using probe of *R. parvifolius* and 50× excess blocking DNA of *R. coreanus* (*R03-6*) (Figures 5 and 6B1).

GISH also provided evidence for our hypothesis: the accession *R03-27* (2*n* = 3*x* = 21) is hybrid origination, from results discriminating parental chromosomes of *R. parvifolius* (*R03-5*) and *R. coreanus* (*R03-6*) (Figures 5 and 6C-E). Using genomic DNA of *R. parvifolius* in the presence of 50× excess unlabelled blocking DNA of *R. coreanus*, GISH revealed 21 hybridization signals in *R03-27*. Fourteen chromosomes almost completely colored with green signals (Figures 5 and 6C1, arrows), while the remained seven had less signals at the centromeric region. When using *R. coreanus* DNA as a probe combined with 50× excess unlabelled blocking DNA of *R. parvifolius*, seven chromosomes were colored by dense signals in most areas (Figures 5 and 6C2, arrows), and the last three had signals in the telomeric regions. All 21 chromosomes of *R03-27* showed strong hybridization signals at centromeric regions without blocking DNA (Figures 5 and 6D). When the ratio of blocking DNA to probe DNA got to 100:1, chromosomes from *R03-27* did not give any signals (Figures 5 and 6E). The GISH of another accession *R03-57* also showed the similar results with that of *R03-27* when using *R. parvifolius* and *R. coreanus* as probes (data not shown in Figures 5 and 6).

## Discussion

### Chromosomal patterns of 45S and 5S rDNA in *Rubus*

In diploid *Rubus* taxa, the observations of two 45S rDNA and two 5S rDNA sites was generally consistent with previous findings in *R. idaeus* and *R. parvifolius* [34-37]. At the diploid level, one set of *Rubus* chromosome (*x* = 7) was typified by the presence of one terminal 45S locus and one proximal 5S locus on the short arm of different chromosomes. 45S rDNA sites localized on the short arm, which might represent the nucleolar organiser regions (NOR) [36,39]. In Rosaceae family, most of the diploid plants examined had two sites of both 45S and 5S rDNA [40-42], and all rDNA exhibited a similar distribution pattern. That is, in general, 45S rDNA repeats resided at the terminal regions, while 5S rDNA sites distributed in interstitial and proximal regions of chromosomes. 5S rDNA sites were also detected at a telomeric (or sub-telomeric) position in some species, such as *Sanguisorba annua* [34], *Fragaria × ananassa* [43] and some *Rubus* taxa in this study. This might be a consequence of chromosome rearrangements [34].

In the three triploid accessions tested, *R03-20* and *R03-27* shared the same number of rDNA sites. But only two 45S and 5S rDNA loci were detected in *R03-57*. The presence of the expected number of 5S sites in the former two might be explained by their recent origin. *R03-57* showed obvious genetic differentiation from its parents during its long evolutionary history, mainly involving chromosomal rearrangement or segment deletion or lost. Of course, other explanations might be reasonable too. It was believable that this site loss has arisen from an early event, as reported among octoploid *Fragaria* and subspecies [44]. The loss of 45S rDNA might be related to nucleolar dominance [45] as well.

Among the six polyploids of subg. *Malachobatus*, multiples of 45S rDNA site number were in proportion to the increasing ploidy level. In the octoploid *R. buergeri* (*R01-11*), there were four times of 45S rDNA loci to that in diploids (Figures 1 and 2R). The site number in the tetraploids was twice as those in diploids (Figures 1 and 2M-P), except *R. setchuenensis*. Higher signal intensity was observed in *R. setchuenensis* (*R01-24*) on one chromosome represented existing of a longer rDNA coding sequence. This might be explaining the possible reason for loss of 45S rDNA by simple translocation and recombination of chromosome segment where 45S rDNA resides. Similar results were recorded in an aneuoctoploid cv ‘Aurora’ [37]. In contrast, the increasing number for 5S rDNA loci was incongruent with the multiplied chromosome number (Figures 1 and 2M-R). For instance, two 5S rDNA sites were detected in both diploid and tetraploid species. In octoploid *R. buergeri* (*R01-11*), chromosome number counted twice of those in tetraploids, but only one additional 5S locus was detected here when compared (Figures 1 and 2R). These results indicated that the duplicated sites of 45S rDNA tend to be conserved, whereas 5S rDNA sites had a tendency toward elimination after polyploidization. This was in agreement with the study in *Fragaria* [44], *Prunus* [46], and *Sanguisorba* [34]. Thus, this tendency might be common in polyploids within Rosaceae.

### Origins of some polyploid *Rubus* taxa

Polyploidy and hybridization are prevalent in *Rubus* [14]. Hybridization in *Rubus* occurs mostly between closely related species [18,47]. The *Rubus* hybrids’ chromosome numbers increased due to fertilization of unreduced gametes from one or both of the parents [48]. Fertilization of unreduced gametes was also considered as a dominant process involving in the origin of polyploid plants by Bretagnolle and Thompson [49].

In this study, we observed the same number, size and distribution patterns of the 45S and 5S rDNA loci in each haploid complement of the diploid *R. parvifolius* (*R03-5*) and triploid *R03-20* (Figure 2D and J). The *R03-20* also showed strong signals at the centromeric region on all chromosomes when using *R03-5* DNA as probes (Figure 5B1). Based on the similarity of rDNA localization as well as in plant morphology but larger leaflet and fruit to the diploid *R. parvifolius* (*R03-5*), we believe the accession *R03-20* was an autotriploid *R. parvifolius*. It was likely that *R03-20* derived from fusion of an unreduced gamete (2*n*) and a reduced gamete (*n*), both from the diploid.

Natural hybridization and introgression has been described as a fundamental evolutionary process of species complexes [50]. It has been reported that *R. parvifolius* and *R. coreanus* could form hybrids, naturally or artificially [18,23]. Together with GISH results, the accession *R03-27* was most likely a natural hybrid between these two species, comprising of an unreduced gamete from *R. parvifolius* and a reduced gamete from *R. coreanus*. Moreover, there were weak hybridization signals in other parts of the chromosome centromere region of *R03-27* (Figures 5 and 6C), indicating the close relationship between its parents. It had also been described in hybrids of *Begonia*, and *Diospyros* species [51,52]. Another accession *R03-57* was also a natural hybrid between *R. parvifolius* and *R. coreanus*.

Allopolyploids have long been recognized as an important mode of plant speciation [53], and it can be identified by 45S rDNA signal number, as well as intensity [41]. Polymorphism of 45S rDNA signal intensities among the polyploids might imply different repeat copy numbers among different rDNA sites [44]. As far as allopolyploid *Rubus* was concerned, Lim et al. [37] reported that one 45S rDNA site was markedly bigger than the rest among the four 45S rDNA sites of the allotetraploid hybrid-berry (R’R’BB, raspberry × blackberry). Here, four (eight) 45S rDNA sites localized on two (four) pairs of different chromosomes in tetraploids (octoploid), with two loci much stronger intensity (Figures 1 and 2M-R). The meiotic pairing of polyploids mostly formed bivalents: with 94% to 98% bivalents for tetraploids; 14.4% univalents, 83.2% bivalents, 0.86% trivalents, and 1.44% quadrivalents for octoploid *R. buergeri* [Nan et al. Studies on meiotic pairing behavior and rDNA distribution pattern in six *Rubus* taxa (Rosaceae), unpublished]. Therefore, we supported the presumption that the six polyploids, *R. lambertianus* var. *glaber* (*R01-8*), *R. parkeri* (*R01-12*), *R. assamensis* (*R01-10*), *R. multibracteatus* (*R01-23*), *R. setchuenensis* (*R01-24*), and *R. buergeri* (*R01-11*), were all of allopolyploidy origin. Moreover, based on the similar rDNA patterns, these tetraploid species probably shared common genomic composition, since they had a common ancestor as proposed by phylogenetic analysis based on different DNA sequences (Alice et al.) [15].

### Implication of *R. parvifolius* in speciation of the two subgenera

The chromosomes were intensely and uniformly labelled with the probes from the same species in GISH analysis, whereas chromosomes were scantily and irregularly labelled with the probes from different species [54]. This could be expected because phylogenetically close species have many DNA sequences in common. Here in a series of GISH experiments, the chromosomes of *R. coreanus* (*R03-6*) hybridized with *R. parvifolius* (*R03-5*) generated the strongest signal intensity. This could be inferred that the genome of *R. coreanus* was most closely related to that of *R. parvifolius* among the species tested. Other taxa in subg. *Idaeobatus*, *R. ellipticus* (*R01-7*) and *R. ellipticus* var. *obcordatus* (*R01-2*) showed close relationship with *R. parvifolius*. It was noteworthy that *R. pinfaensis* (*R01-22*) was also close to *R. parvifolius* even though they were assigned to two different sections. The signal strength was varied among different taxa (Figures 3 and 4A-I), indicating the different repeat number of DNA sequences among them. Our previous GISH results showed that *R. coreanus* had remote genetic relationship with *R. tsangii* and *R. corchorifolius* [55]. In this study, it seemed that *R. parvifolius*, rather than *R. coreanus*, had more close genetic relationship with these taxa in subg. *Idaeobatus*.

In the five tetraploids tested from subg. *Malachobatus*, GISH results also suggested that to whom it was *R. parvifolius* (*R03-5*) rather than *R. coreanus* (*R03-6*) that had much closer relationship with them. GISH signals showed that the common sequences with *R. parvifolius* concentrated not only at the telomere, but also at the centromere of four to fourteen chromosomes (Figures 3 and 4J-N). When using *R. coreanus* DNA as a probe, only two signals at telomeric region were detected in tetraploid *R. assamensis* (*R01-10*) (Figures 3 and 4O). The variation in GISH signal intensity, produced by probes of *R. parvifolius* and *R. coreanus* (Figures 3 and 4), reflected the close relationship of these tetraploid species with *R. parvifolius*, yet distant with *R. coreanus*.

*Rubus parvifolius* was more genetically variable when compared with *R. coreanus* [13,56]. Extensive crossing and formation of natural hybrids with many species from both subgenera *Idaoebatus* and *Malachobatus* were found in *R. parvifolius* [18,19,21-23]. The fact that genomic DNA of *R. parvifolius* (*R03-5*) generated several common repetitive DNAs in polyploid species (Figures 3 and 4J-O) here again highlighted the important role of *R. parvifolius* in hybridization, polyploidization and speciation of the two subgenera. Therefore it is likely just the crossability of such species as *R. parvifolius* that account for the gene flow or introgression in the genus.

## Conclusions

In summary, the duplicated 45S rDNA sites tend to be conserved, whereas those of 5S rDNA tend to be eliminated after polyploidization in the genus *Rubus*. The signals from FISH indicated that the polyploids tested in this study are allopolyploid origin. Reciprocal GISH analysis between *R. parvifolius* and polyploids reveals diverse signal number and distribution patterns, and the important role of *R. parvifolius* in hybridization, polyploidization and speciation of the two subgenera is highlighted. However, there is not enough evidence revealing phylogenetic relationships between the subg. *Idaeobatus* diploid species and *Malachobatus* polyploid species in *Rubus* from this study. To further elucidate the phylogeny within *Rubus*, molecular data produced by multiple DNA sequences as well as morphological evidence with more species are necessary.

## Methods

### Plant materials

18 accessions from 9 sections of subgenera *Idaeobatus* and *Malachobatus* were used in this study (Table 1), including 14 *Rubus* taxa (9 diploids, 5 tetraploids and an octoploid) and 3 resembling *R. parvifolius* accessions (3 triploids). All plants were planted in the Teaching and Scientific Research Base of Sichuan Agricultural University. The voucher specimens were deposited in the herbarium of the College of Horticulture, Sichuan Agricultural University, China.

### Chromosome preparations

Chromosome preparation was followed the procedures of Wang et al. [13]. Briefly, root tips from cutting propagation canes were pretreated in 0.002 mol · L^−1^ 8-hydroxyquinoline at 4°C for 4 h, and fixed in Carnoy’s I solution (absolute ethanol : glacial acetic acid = 3 : 1, v/v) at 4°C for about 24 h. The fixed root tips were hydrolyzed in 1 mol · L^−1^ HCl at 60°C for 30-40 s, stained with Carbol Fuchsin, and then squashed with 45% acetic acid. The chromosome slides with well-spread metaphases were frozen in liquid nitrogen for 5 min. After removal of the coverslip, slides were air dried, and then kept at -80°C until use.

### DNA extraction and probe preparation

Total genomic DNA was isolated from 0.1 g unexpanded leaf tissue using a modified CTAB protocol [57]. 45S rDNA probe was labelled with biotin-16-dUTP using nick translation (Roche Applied Science, Mannheim, Germany) using primers 18S-F1 (5’-TAC CTG GTT GAT CCT GCC AGT A-3’) and 18S-R1 (5’-CAA TGA TCC TTC CGC AGG TTC A-3’) [58] with DNA template from *R. coreanus*. 5S rDNA probe was amplified and directly PCR-labelled by digoxigenin probe synthesis kit (Roche) with primers 5S1 (5’-GGA TGC GAT CAT ACC AG CAC-3’) and 5S2 (5’-GGG AAT GCA ACA CGA GGA CT-3’) [59].

For GISH, the genomic DNA were labelled by a nick-translation reaction with digoxigenin-11-dUTP (Roche). *R. parvifolius* (*R03-5*; 2*x*), *R. coreanus* (*R03-6*; 2*x*) and *R. assamensis* (*R01-10*; 4*x*) DNA were used in GISH analysis (Figures 3, 4, 5 and 6).

### *In situ* hybridization

*In situ* hybridization was performed with minor modifications of the procedure by Lim et al. [37]. Prior to hybridization, the chromosome slides were treated with an enzymatic mixture (2% Cellulase and 2% Pectinase, w/v, Sangon, China) at 37°C for 1.5 h, followed by incubating in 100 μg · mL^−1^ RNase A (Sigma-Aldrich, St Louis, MO, USA) solution (in 2 × SSC, saline sodium citrate buffer) at 37°C for 1 h. Afterwards, they were digested with 1 mg · mL^−1^ Proteinase K (Sangon, China) at 37°C for 30 min, fixed in 4% (w/v) paraformaldehyde at room temperature (RT) for 10 min, and dehydrated in an increasing series ethanol of 75%, 95%, 100% for 5 min each. The slides were denatured with 70% (v/v) formamide in 2 × SSC at 70°C for 3 min and dehydrated immediately using an ice-cold ethanol series (75%, 95% and 100%) for 5 min, respectively. The hybridization mixture contained ~100 ng labelled of probe, 2 × SSC, 50% (v/v) formamide deionized, 10% (w/v) dextran sulfate (DS), 0.1% (v/v) sodium dodecyl sulfate (SDS) and 300 ng sheared salmon sperm DNA or unlabelled blocking DNA. The ratio of blocking DNA to probe DNA was 0, 50:1, or 100:1 in hybrid identification (Figures 5 and 6).

The hybridization mixture was denatured at 100°C for 5 min and placed instantly on ice for 10 min. 30 μL of the hybridization mixture was applied to each slide, and then hybridized overnight at 37°C in a humid chamber. The stringency conditions were decided by the concentration of formamide in hybridization mixture together with the condition of post-hybridization washing according to the calculations by Schwarzacher and Heslop-Harrison [60]. Post-hybridization washes were performed in 20% (v/v) formamide (in 0.1 × SSC) at 42°C for 10 min, and 2 × SSC at RT for 5 min, which resulted in a comparatively high stringency (~85%). Biotin-labelled and digoxigenin-labelled probes were detected with tetramethyl-rhodamine isothiocyanate (TRITC) (Thermo) and avidin-fluorescein isothiocyanate (FITC) (Roche) in 0.5% (w/v) BSA (bovine serum albumin) solution (in 1 × PBS, phosphate buffer saline), respectively. The chromosome slides were counterstained with 2 ng · μL^−1^ 4’, 6-diamidino-2-phenylindole (DAPI, Sigma) in the VectaShield antifade solution (Vector Laboratories, Burlingame, California, USA).

The images were captured with a high-resolution cooled CCD camera using a fluorescence microscope (Olympus BX 51, Japan), and processed by Image Pro-Plus 6.0 (Openlab, Improvision, UK). A color composite image was merged by using the Color Composite feature with multiple fluorescent images acquired as monochrome single wavelengths. At least five mitotic metaphase complements per accession were scored. The karyotypes of FISH and GISH were referenced and followed our previous results as Wang et al. [12,13]. The cytological classification of somatic metaphase chromosomes follows the categories of Tanaka [61,62].

## Abbreviations

DAPI: 4’, 6-diamidino-2-phenylindole
DIG: Digoxigenin
FISH: Fluorescence *in situ* hybridization
FITC: Avidin-fluorescein isothiocyanate
GISH: Genomic *in situ* hybridization
rDNA: Ribosomal DNA
rRNA: Ribosomal RNA
TRITC: Tetramethyl-rhodamine isothiocyanate
PCR: Polymerase chain reaction
